# Insights into the Hormone-Regulating Mechanism of Adventitious Root Formation in Softwood Cuttings of *Cyclocarya paliurus* and Optimization of the Hormone-Based Formula for Promoting Rooting

**DOI:** 10.3390/ijms25021343

**Published:** 2024-01-22

**Authors:** Yuan Tian, Wanxia Yang, Shiying Wan, Shengzuo Fang

**Affiliations:** 1 College of Forestry and Grassland, Nanjing Forestry University, Nanjing 210037, China; tianyuan@njfu.edu.cn (Y.T.); yanwanxia@njfu.edu.cn (W.Y.); wsy9807@163.com (S.W.); 2 Co-Innovation Center for Sustainable Forestry in Southern China, Nanjing Forestry University, Nanjing 210037, China

**Keywords:** wheel wingnut, rooting parameters, hormone signaling pathway, structural gene expression, transcriptome, WGCNA

## Abstract

Adventitious root (AR) formation is vital for successful cutting propagation in plants, while the dynamic regulation of phytohormones is viewed as one of the most important factors affecting AR formation. *Cyclocarya paliurus*, a hard-to-root plant, is faced with the bottleneck of cloning its superior varieties in practice. In this study, ten treatments were designed to figure out the best hormone-based formula for promoting AR formation in softwood cuttings and explore their hormone-regulating mechanisms. Both the rooting process and the rooting parameters of the softwood cuttings were significantly affected by different hormone-based formulas (*p* < 0.05), while the greatest rooting rate (93%) and root quality index were achieved in the H3 formula (SR3:IR3 = 1:1). Significant differences in the measured phytohormone concentrations, as well as in their ratios, were detected among the cuttings sampled at various AR formation stages (*p* < 0.05), whereas the dynamics for each phytohormone varied greatly during AR formation. The transcriptome analysis showed 12,028 differentially expressed genes (DEGs) identified during the rooting process of *C. paliurus* cuttings, while the KEGG enrichment analysis indicated that a total of 20 KEGG terms were significantly enriched in all the comparison samples, with 253 DEGs detected in signal transduction. Furthermore, 19 genes with vital functions in regulating the hormone signaling pathway were identified by means of a WGCNA analysis. Our results not only optimize a hormone-based formula for improving the rooting of *C. paliurus* cuttings but also provide an insight into the hormonal regulatory network during AR formation in softwood *C. paliurus* cuttings.

## 1. Introduction

Wheel wingnut (*Cyclocarya paliurus*), a sole species in the genus, belongs to the Juglandaceae family and is a multi-functional tree species [1]. Although it is now only distributed across subtropical China, *Cyclocarya* has a long fossil record in North America, Europe, and eastern Asia and is a paleoendemic Chinese genus that went extinct in North America and Europe during the Cenozoic period [2,3]. With their high content of flavonoids, triterpenoids, and other bioactive metabolites, *C. paliurus* leaves are widely used for the extraction of pharmaceutical and nutraceutical ingredients [4,5,6]. Since *C. paliurus* leaves were listed as new food raw materials by the National Health and Family Planning Commission of China in 2013 [3], many of the plant’s natural forests have been destroyed to harvest its leaves. Thus, the best option for *C. paliurus* leaf production, as well as for protecting its natural forests, is to develop *C. paliurus* plantations using the plant’s superior genotypes in suitable sites.

The accumulation of bioactive metabolites in plants are determined by both internal genetic control and external environmental factors. It has been reported that the contents of bioactive metabolites significantly vary among the different genotypes of *C. paliurus* [1,7], whereas several superior genotypes have been selected and bred for the leaf production of bioactive metabolites [8,9]. In order to maintain the excellent traits of these superior genotypes, asexual reproduction is a feasible strategy for propagating uniform seedlings for establishing *C. paliurus* plantations. It has been reported that graft union death, scion–rootstock compatibility, and labor-intensive grafting techniques influence the success and survival of black walnut plants (*Juglans nigra*) [10,11]. Our previous study indicated that grafting, as a routine method of asexual propagation, is also not ideal for *C. paliurus* because optimum grafting survival only reached 53.5% [12]. Therefore, propagation via softwood and hardwood cuttings has been extensively investigated to find an ideal alternative grafting method as well as quickly replicate desired clones of *C. paliurus* [13,14]. As a hard-to-root woody plant, however, the rooting rate and rooting quality of *C. paliurus* achieved via cutting propagation are unable to meet the requirements of large-scale production, despite the method’s potential value in commercial production. 

Adventitious root (AR) formation, the prerequisite for successful cutting propagation, is the bottleneck for the survival of isolated cuttings [15], involving a multistage, multilevel development process. During AR formation, cuttings undergo initial expansion, callus formation, and rooting stages [11,14]. Correspondingly, the specific cells at the wound mainly experience induction stages, reprogram into cells with the ability to form adventitious roots, and enter a continuous division process towards initial primordia and callus formation, and, finally, the roots break through the callus [16]. The whole AR formation process is thought to be the response of cuttings to certain environmental stimuli, mainly including wounding responses at the cutting site and signal network isolation from the whole plant [17,18]. However, many studies indicated that the dynamic regulation of phytohormones is one of the most vital endogenous factors affecting AR formation [17,19,20].

Phytohormones, such as auxin, cytokinin, gibberellin, and abscisic acid, play crucial roles in the regulation of AR formation [21,22]. Recent research has indicated that auxin can regulate AR formation by directing cell division, elongation, and differentiation [23]. Both indole-3-acetic acid (IAA) and indole-3-butyric acid (IBA) are the most important natural auxins in plants. IAA is the central player, as a promoter, in AR formation [21,24,25], while IBA plays an important role in the induction stage. Therefore, a rooting promoter with IBA as the main component is often used to induce adventitious root formation and improve the rooting rate of cuttings, especially for hard-to-root species [26]. Moreover, it is also reported that cytokinin (CK) can positively regulate cell dedifferentiation, shoot formation, and lateral root development [27,28,29], whereas the ratio of auxin to cytokinin has been demonstrated to be closely related to competence in AR formation [22,27,28]. Moreover, gibberellin (GA) and abscisic acid (ABA) are widely acknowledged to be barriers to AR formation, and low levels of GA and ABA are beneficial for AR formation in cuttings [30,31]. 

It is reported that phytohormones regulate the AR formation process by providing inherent signaling for determining cell fates and responding to stresses [17]. In recent years, some studies tried to reveal the phytohormone regulatory network using transcriptomic analyses and RNA-seq technology, especially focusing on the key regulatory components involved in the hormone signaling pathway [17,32,33]. For instance, the key transcription factors of auxin, CK, GA, and ABA signaling have been well investigated, and it has been suggested that AR formation in some plants is controlled by coordinated hormonal regulation via a series of complex interactions [21,27]. Auxin, as the core of the cross-regulatory network [29], has confirmed that interactions of auxin signaling with CK, GA, and ABA exist, as seen in the studies of Arabidopsis roots [15,32,34], but the phytohormone regulation network in woody plants, such as *C. paliurus*, is still poorly understood during AR formation. In this study, our objectives were as follows: (1) figure out the best hormone-based formulas to promote AR formation in softwood cuttings of *C. paliurus*; (2) explore the relationship between the endogenous hormone dynamic and the AR formation of *C. paliurus* softwood cuttings; and (3) screen the key genes involved in hormonal regulation and construct an auxin cross-regulatory network. The results from this study can not only provide insights into the molecular mechanisms taking place during the AR formation process in *C. paliurus* but also afford technical support for other hard-to-root tree species reproduced via cutting propagation.

## 2. Results

### 2.1. Variations in the Rooting Process and Related Rooting Parameters

**Figure 1:**
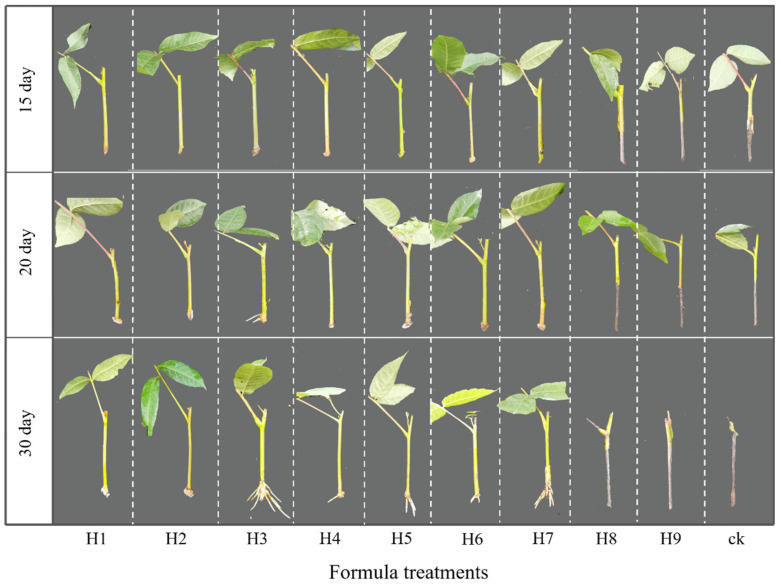
Effects of different hormone-based formulas on the characteristics of adventitious root formation in softwood cuttings of *C. paliurus* at different sampling times. H1, H2, H3, H4, H5, H6, H7, H8, and H9 represent different hormone-based formula treatments, while ck denotes a control group of *C. paliurus* soft cuttings treated using ABT-1 alone.

The different formulas obviously affected the rooting process of the softwood cuttings (Figure 1 and Figure S1). For example, after 15 days of cuttage, all cuttings entered the initial expansion stage apart from the treatments of ck, H1, H8, and H9. However, one-third of the cuttings in the H3 treatment were at the rooting stage after 20 days of cuttage, whereas most of the cuttings were still at the callus formation stage in the other treatments. Notably, about half of the cuttings in the ck, H8, and H9 treatments were at the initial expansion stage, while about 5% and 50% of the cuttings in H8 and ck tended to be blackened and rotted at the cutting base, respectively. However, after 30 days of cuttage, the majority of cuttings in the H3 treatment, half in the H7 treatment, and one-third of cuttings in the H4 and H6 treatments were at the rooting stage, respectively (Figure S1). It is worth noting that all the cuttings in the ck treatment, as well as 33% of the cuttings in the H8 and H9 treatments, have been blackened and rotted at the cutting base (viewed as dead).

**Figure 2:**
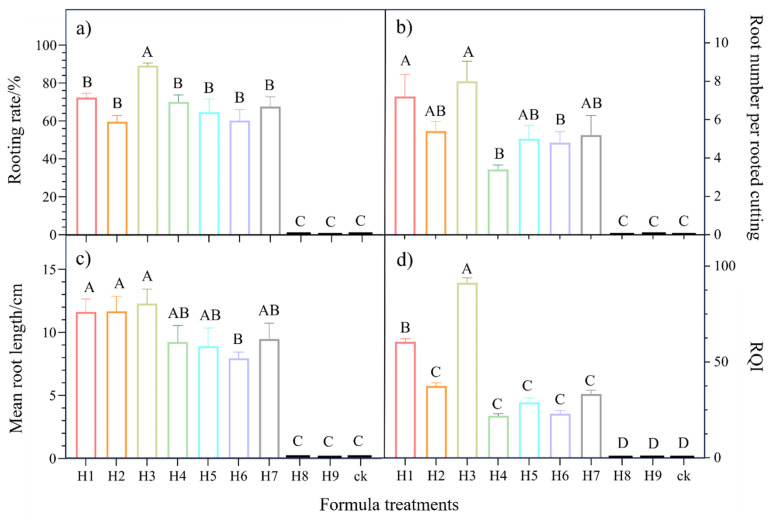
The effects of different hormone-based formulas on the rooting rate (**a**), rooting parameters (**b**,**c**), and rooting quality indexes (RQIs, (**d**) of *C. paliurus* soft cuttings at 60 days after treatments. Different capital letters indicate a significant difference among the treatments at *p* < 0.05 for each measured parameter. H1, H2, H3, H4, H5, H6, H7, H8, and H9 represent different hormone-based formula treatments, while ck denotes a control group of *C. paliurus* soft cuttings treated using ABT-1 alone.

The rooting rates ranged from 0.0 to 93.0% after 60 days of treatment (Figure 2a). The one-way ANOVA indicated that there was a significant difference in rooting rate among different treatments (*p* < 0.05), with the greatest rate of 93% in the H3 treatment and the lowest (no rooting) in the H8, H9, and ck treatments. Our results showed that the formulas with the ABT-1 (containing NAA) addition were unfavorable to improving the rooting rate of *C. paliurus* softwood cuttings, whereas the addition of IR3 and SR3 could promote the rooting rate of the cuttings.

Similarly, the root number per rooted cutting (Figure 2b), mean root length (Figure 2c), and root quality indexes (RQIs, Figure 2d) were also significantly affected by the various formula treatments (*p* < 0.05). For instance, the RQI values among the different treatments were ranked as H3 > H1 > H2 > H7 > H5 > H6 > H4 > H8 ≧ H9 ≧ ck. The RQI reached 91.36 in the H3 treatment, which is 30.79, 53.81, 58,04, 62.51, 68.36, and 69.37 greater than that in the H1, H2, H7, H5, H6, and H4 treatments, respectively.

### 2.2. Dynamics of Endogenous Hormones during the AR Formation Process

**Figure 3:**
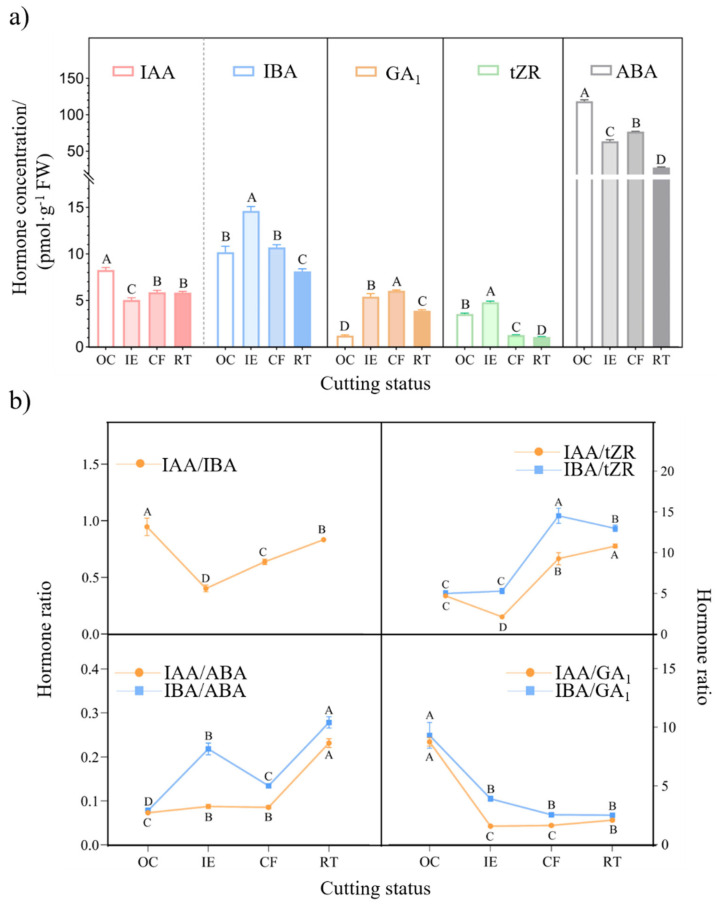
The dynamics of phytohormone concentrations and the ratios of auxin to other phytohormones in *C. paliurus* soft cuttings during AR formation. (**a**) The concentration dynamics of auxin (IAA and IBA), cytokinin (tZR), gibberellin (GA_1_), and abscisic acid (ABA). (**b**) Dynamics in the ratios of auxin (IBA and IAA) to other phytohormones (tZR, GA_1_, and ABA represent cytokinin, gibberellin, and abscisic acid, respectively). Different capital letters indicate statistically significant variations among the AR formation status for the same phytohormone (*p* < 0.05). OC: original cutting; IE: cuttings in the initial expansion stage; CF: cuttings in the callus formation stage; and RT: cuttings in the rooting stage.

Significant differences existed in the concentrations of measured phytohormones among the cuttings sampled at various stages (Figure 3a, *p* < 0.05). In terms of auxin concentration, the IBA level (ranging from 15.17 pmol·g^−1^ to 5.84 pmol·g^−1^) was always higher than the IAA level (ranging from 8.28 pmol·g^−1^ to 5.05 pmol·g^−1^) in the cuttings sampled at four stages. However, the highest IAA and IBA concentrations were detected in the original cutting (OC) and initial expansion cutting (IE), respectively. Compared to the OC, the IBA concentration of the IE increased by 43.7% but decreased by 20.2% in the rooting cutting (RT), indicating that IBA plays a key role in the initial expansion of *C. paliurus* soft cuttings. 

The dynamics of the CK (tZR) and GA (G”_1_) concentrations were similar during the AR formation process. However, the tZR concentration reached its peak (4.81 pmol·g^−1^) in the IE, whereas the GA_1_ concentration reached its highest level (5.95 pmol·g^−1^) in the callus formation cutting (CF). It was noted that the ABA concentrations in the *C. paliurus* soft cuttings were much greater than the other phytohormone concentrations during AR formation. However, the ABA concentration in the IE, CF, and RT decreased by 46.5%, 35.2%, and 76.6%, respectively, when compared with the OC, showing a decreasing trend during the AR formation process.

The ratio of IAA to IBA (IAA/IBA) in the cuttings dropped sharply during the initial expansion stage (from 0.95 to 0.40), and then significantly increased in the following stages (Figure 3b). Both the IAA/tZR and IBA/tZR ratios showed an upward trend during the AR formation of *C. paliurus* soft cuttings, especially in the CF. Compared to the OC, the ratios of IAA to tZR (IAA/tZR) in the CF and RT enhanced by 97% and 130%, respectively, while the IBA/tZR values increased by 192% and 160%, respectively. Similarly, an overall upward trend was also observed for the auxin/ABA ratio during the AR formation process. However, it is worth noting that the IBA/ABA ratio increased from 0.07 in the OC to 0.22 in the IE, but significantly dropped to 0.13 in the CF, undergoing a more intensive change. In contrast, the ratios of auxin to GA_1_ (IAA/GA_1_ and IBA/GA_1_) showed a downward trend during the AR formation process. For example, the IBA/GA_1_ ratios in the IE, CF, and RT decreased by 58%, 72%, and 73%, respectively, when compared with the value in the OC.

### 2.3. Overall Assessment of Transcriptome Profiles during the AR Formation Process

**Figure 4:**
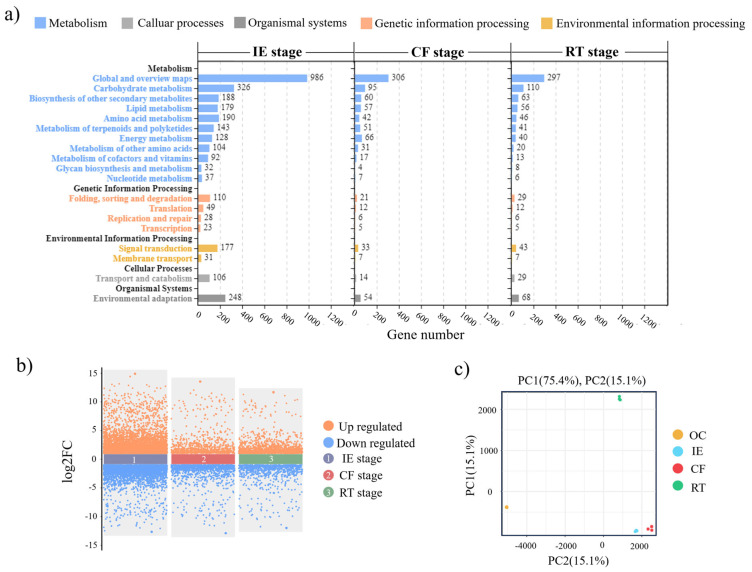
KEGG pathway annotations and PCA score plot with transcriptional profiles of *C. paliurus* soft cutting samples, and the variation of up-regulated and down-regulated DEGs during the AR formation of *C. paliurus* soft cuttings. (**a**) KEGG pathway annotations of the statistic map with transcriptional profiles of all samples. (**b**) The variation of up-regulated and down-regulated DEGs in different stages of AR formation. (**c**) PCA score plot: each point in the PCA score plot represents an independent biological replicate. OC: original cutting; IE: cuttings in the initial expansion stage; CF: cuttings in the callus formation stage; and RT: cuttings in the rooting stage. IE stage: initial expansion stage; CF stage: callus formation stage; and RT stage: rooting stage.

Using RNA-seq technology, 165,171,744,300 bp of raw data were generated. After filtering, 163,774,529,712 bp of clean data were mapped to the reference transcriptome (Table S1), and the average mapping ratio was 90.3% (Table S2). A total of 12,028 expressed genes were detected in all the samples. However, 7802 DEGs (4481 up-regulated and 3321 down-regulated) were detected in the IE stage, while 1955 DEGs (826 up-regulated and 1129 down-regulated) were found in the CF stage. In the RT stage, 2271 DEGs were detected, where 1452 were upregulated and 819 were downregulated (Figure 4b). PCA results showed that PC1 and PC2 explained 74.5% and 15.1% of the variation in gene expression among all the samples, respectively (Figure 4c). 

Based on the KEGG database, the functions of the DEGs were divided into five categories: metabolism, cellular processes, organismal systems, genetic information processing, and environmental information processing (Figure 4a). Furthermore, a total of 20 KEGG terms were significantly enriched in all the comparison samples, whereas the DEGs were mainly enriched in carbohydrate metabolism, environmental adaptation, and signal transduction. These results suggested that although active carbohydrate metabolism is fundamental for the AR formation process as a nutritional supplement after cuttage, signal transduction, specifically plant hormone signaling, plays a vital role in all stages of AR formation via efficiently inducing cell reprograming, enabling cells to obtain a new evolutionary pattern and develop towards AR formations [18].

### 2.4. DEGs Involved in Phytohormone Signal Transduction Pathways

**Figure 5:**
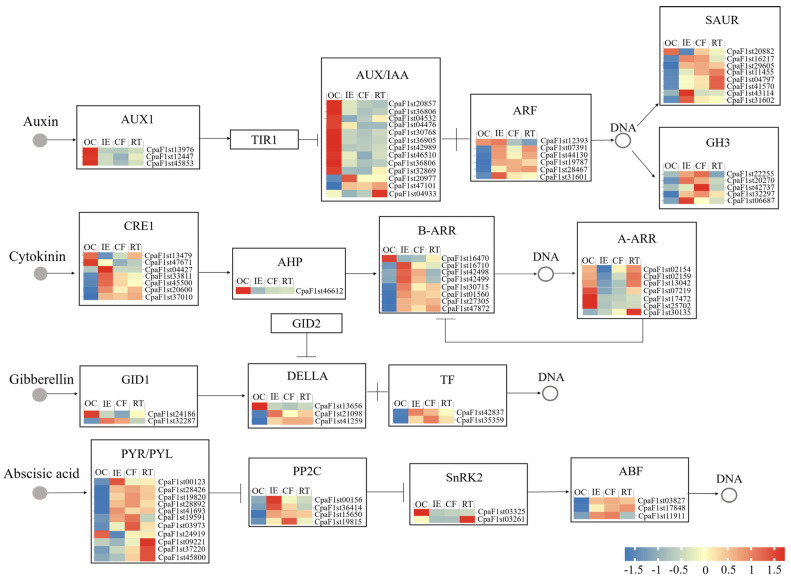
The expression profiles of the phytohormone-related genes in *C. paliurus* soft cuttings during AR formation. Red indicates up-regulated genes, and blue indicates down-regulated genes. OC: original cutting; IE: cuttings in the initial expansion stage; CF: cuttings in the callus formation stage; and RT: cuttings in the rooting stage.

In order to figure out the regulatory mechanism of phytohormones in the AR formation of *C. paliurus* soft cuttings, the DEGs assigned to the key components involved in the phytohormonal signal transduction pathways were identified (Figure 5). In the auxin signaling pathway, 35 DEGs were detected, and we found that most of the *LAX*s, *AUX*s, and *IAA*s were down-regulated, but most auxin response genes, *ARF*s and *GH3*s, as well as *SAUR*s in auxin downstream signaling, were up-regulated during the AR formation process. In the CK signaling pathway, 23 DEGs were detected, among which the CRE1-related genes and *B-ARR*s were up-regulated at the IE stage but down-regulated in the following stages. Meanwhile, the majority of *A-ARR*s were up-regulated in the IE and RT stages but down-regulated in the CF stage. In addition, seven DEGs were attached to the GA signaling pathway, where the DELLA-related and TF-related genes were up-regulated in the IE stage while they were down-regulated in the following stages. A total of 20 DEGs were also detected in the ABA signaling pathway; however, most of the *PYL*s, *PP2C*s, and *ABF*s in the ABA signal transduction pathway were up-regulated in the IE stage while they were down-regulated in the CF and RT stages.

### 2.5. Co-Expression Network Analysis of Weight Genes

**Figure 6:**
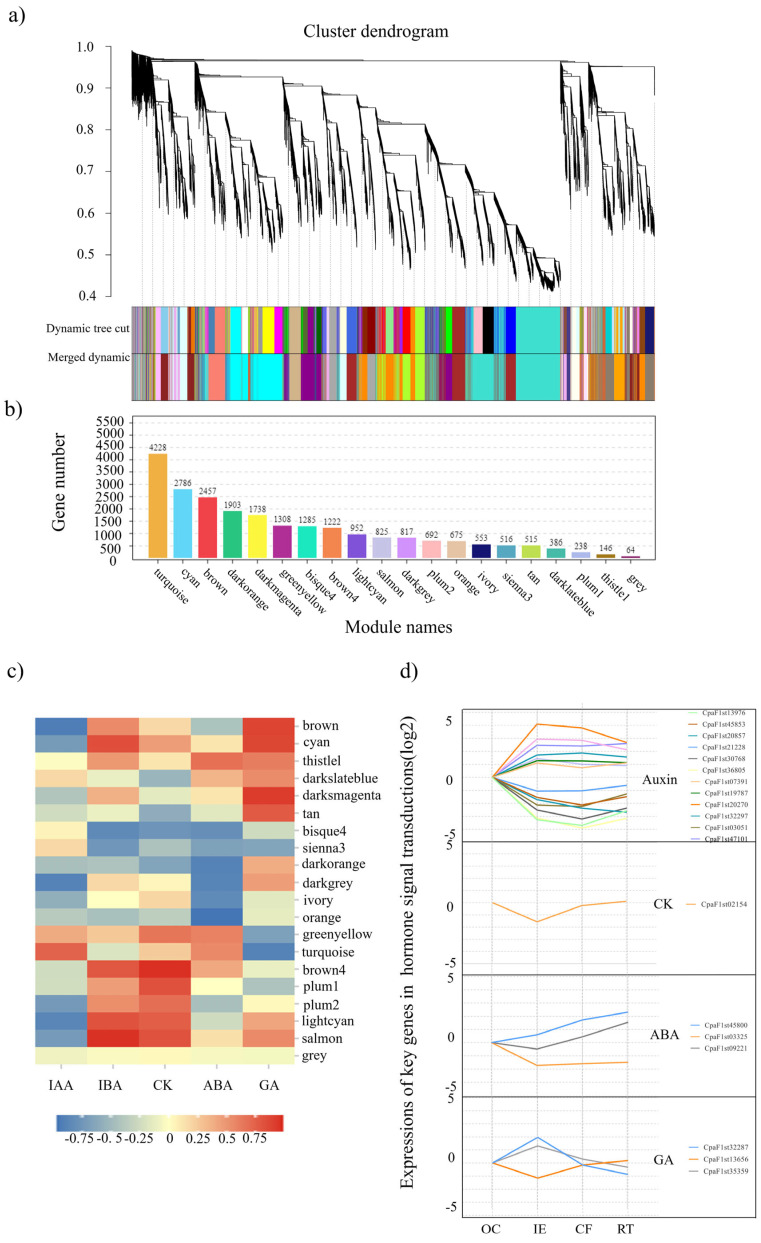
The results of the gene co-expression network analysis. (**a**) A hierarchical cluster tree identified by means of a WGCNA. Hierarchical cluster tree showing co-expression modules, while the major tree branches constitute 20 modules labeled using different colors. (**b**) The number of genes in each module. (**c**) The heatmap of correlation coefficients between phytohormone dynamics and module eigengenes, with the red and blue blocks representing positive and negative correlations, respectively. (**d**) Key genes involved in hormonal regulation identified by the WGCNA analysis and their expression patterns. CK, GA, and ABA represent cytokinin, gibberellin, and abscisic acid, respectively. OC: original cutting; IE: cuttings in the initial expansion stage; CF: cuttings in the callus formation stage; and RT: cuttings in the rooting stage.

Based on the dynamics of the phytohormone concentrations measured and all the DEGs identified in all the collected samples, a weighted gene co-expression network analysis (WGCNA) was performed. A total of 20 distinct modules (defined as highly interconnected gene clusters and marked with different colors) were identified (Figure 6a,b), while the modules of the top five highest correlations with each phytohormone were figured out by means of the module sample trait correlation analysis (Figure 6c and Table S3). In terms of the gene significance (GS) values obtained, 12 genes were selected as the key regulatory genes in auxin signal transduction., e.g., *LAX*s (CpaF1st13976 and CpaF1s45853), *AUX*/*IAA*s (CpaF1st20977 and CpaF1st21218.etc), *ARF3* (CpaF1st07391), *ARF9* (CpaF1st19787), *GH3.6* (CpaF1st32297), and *SAUR23* (CpaF1st03051). Similarly, *ARR4* (CpaF1st21054) was recommended as the key regulatory gene in the cytokinin signaling pathway. Moreover, three genes, i.e., *PYL*s (CpaF1st09221 and CpaF1st45800) and *SnRK2* (CpaF1st03325), were identified as the key regulatory genes in the ABA signaling pathway, while a total of three genes, i.e., *GID1* (CpaF1st 32287), *GAIPB* (CpaF1st13656), and *PIF1* (CpaF1st35359), were regarded as the key regulatory genes in GA signal transduction during the AR formation of *C. paliurus* soft cuttings (Figure 6d and Table S4). 

Additionally, a total of 41 genes might be involved in hormonal interactions (Table S5), among which 11 genes might be involved in the interactions between auxin and CK, i.e., *ARR9*s (CpaF1st17472, CpaF1st25702, and CpaF1st17472), *AHP* (CpaF1st33811), and *IAA11* (CpaF1st36927), were strongly correlated with the interactions between IAA and CK, while *AARR4*s (CpaF1st21054 and CpaF1st21059) are highly correlated with the interactions between IBA and CK. Meanwhile, 15 genes were detected as being highly related to the crosstalk between auxin and gibberellin, i.e., *IAA*s (CpaF1st32869, CpaF1st21228, and CpaF1st30768), *AUX22D* (CpaF1st36805), *GH3.6* (CpaF1st32297), and DELLA-related genes (CpaF1st32297and CpaF1st21098) may jointly regulate the crosstalk between IAA and GA, and *GH3.6* (CpaF1st20270) and *GIF1B* (CpaF1st32287) might mediate the interactions between IBA and GA. Moreover, 15 genes were screened out that were assigned to the interaction between auxin and ABA, i.e., *ARF3* (CpaF1st07391), *ARF9* (CpaF1st28476), *IAA14* (CpaF1st04476), *PYL*s (CpaF1st28892, CpaF1st 41693, and CpaF1st19820), and *ABF*s (CpaF1st03827 and CpaF1st11911) may regulate the interactions between IAA and ABA, whereas *PYL4* (CpaF1st09221) and *PP2C06* (CpaF1st36414) likely affected the interactions between IBA and ABA.

### 2.6. qRT-PCR Verification for Selected Genes

To validate the gene expression data from the RNA-seq analysis, nine genes were randomly selected from the transcriptome data for qRT-PCR validation. The results showed that these two types of expression data were highly consistent, with an R^2^ value all over 0.79 (Figure S2), suggesting that the sequencing results were very reliable.

## 3. Discussion

### 3.1. Effects of Different Hormone-Based Formulas on Rooting Efficiency

Plants in the Juglandanceae family, such as *C. paliurus* and *Carya illinoinensis*, have hard-to-root characteristics in their cuttings [13,35]. In recent years, some studies have indicated that the application of suitable exogenous hormones and hormonal formulas could improve the rooting efficiency of hard-to-root tree species [13,35], but the effects of each hormone on the rooting efficiency are species-specific. For instance, Zhang et al. [35] reported that treating the base of *C. illinoinensis* hardwood cuttings by 0.09% NAA and 0.06% IAA could improve the rooting rate up to 82% and 80% with 8.3 and 6.8 roots per rooted cutting, respectively, whereas Guo et al. [36] indicated that the highest rooting rates were only 10.5% and 23.2% after soaking the base of *C. paliurus* cuttings with different concentrations of NAA and IAA, respectively. Indeed, many studies on *C. paliurus* cutting propagation have been conducted to enhance the rooting rate [8,13,14,26,36]; however, the existing results cannot meet the requirements for a large-scale reproduction. For example, the greatest rooting rate (58.0%) for the soft *C. paliurus* cuttings was obtained under the treatment of a 200 mg·L^−1^ GGR solution, with an average root number of 7.2 per rooted cutting [26], whereas the highest rooting rate (44.1%) was achieved for the hard *C. paliurus* cuttings treated with the formula of IR3 and SR3 mixed powder [14]. Thus, improving the rooting efficiency of *C. paliurus* cuttings is an urgent need in the practice for the quick propagation of its superior genotypes.

Our study showed that hormone-based formulas with SR3 and IR3 additions (such as H1, H2, and H3) significantly increased the rooting rate, average root length, average number of roots, and root quality index of *C. paliurus* soft cuttings. Encouragingly, the rooting rate treated with the H3 (SR3:IR3 = 1:1) hormone-based formula reached 93%, and the days for entering the rooting stage were shortened to 20 days after cuttage, providing a feasible solution for cutting propagation of other hard-to-root tree species. However, the cuttings treated with the H7, H8, and H9 formulas with ABT-1 addition were not conducive to AR formation, in agreement with the results reported by Zhang [37] in that soaking the soft cuttings of *C. paliurus* with various ABT-1 concentrations only achieved about a 10.0% rooting rate. It seems that NAA application may not promote the AR formation of *C. paliurus* soft cuttings.

### 3.2. Relationship between Endogenous Hormones and AR Formation

The formation of adventitious roots is a complex process of organ regeneration involving multiple hormonal interactions [21,29]. It was reported that IAA is the main hormone that promotes root primordial differentiation and adventitious root elongation in the AR formation of woody species, such as mulberries [38], *Platycladus orientalis* [39], and *C. illinoensis* [40]. Some studies showed that IAA would accumulate at the base of cuttings after cuttage due to the polar transport of auxin [23,41]. However, our study indicated that the IAA concentration in the *C. paliurus* cuttings obviously decreased after cuttage, whereas the IBA concentration significantly enhanced, especially at the initial expansion stage (Figure 4a), confirming that IBA plays an important role in the inducing stage. Although IBA is a sort of endogenous storage auxin that can be converted into IAA under certain conditions, the IBA–IAA conversion ability is limited for the hard-to-root species [21]. Therefore, there may exist an optimum trade-off between IAA and IBA during the AR formation process, and this trade-off would be species-specific as well as stage-specific. For instance, compared to the IE stage, IAA gradually accumulated in the cuttings during the CF and RT stages, but IBA decreased, thus promoting the AR formation of *C. paliurus* soft cuttings. 

Our study also found that the CK (tZR) concentration in *C. paliurus* soft cuttings increased significantly at the IE stage (Figure 3a) and then declined, which is consistent with some previous studies [20,41]. Our results confirmed that a high concentration of cytokinin is conducive to the dedifferentiation of cells reprogramed to initial root primordium cells in the early stage after cuttage, while the high cytokinin concentration inhibits the formation of adventurous roots after the induction stage. Meanwhile, a downward trend in the ABA concentration was detected during the AR formation process (Figure 3a), suggesting that a continuous decrease in the ABA level should be beneficial for the formation of adventitious roots, in agreement with the results from Liu et al. [39] and Zhang and Wang [42]. However, the role of GA in the AR formation process is still controversial. For example, some studies indicated that GA had an inhibitory effect on the AR formation process in the cuttings of catalpa [43], *Pseudostellaria* [44], and poplars [45], whereas it could promote the initiation and extension of adventitious roots in deepwater rice [46]. Based on the dynamics of GA_1_ level during the AR formation of *C. paliurus* soft cuttings (Figure 3a), we speculate that GA_1_ plays different roles in different stages during the AR formation process of *C. paliurus* soft cuttings, i.e., a higher concentration of GA_1_ may promote the formation of root primordia in the IE stage, while a lower concentration of GA_1_ is beneficial for root elongation in the RT stage.

It is widely believed that AR formation is also regulated by hormonal interactions and their trade-offs, while auxin is the central node of hormone regulation [21,27,39]. The ratios of auxin to other hormones have been demonstrated to be closely related to competence in AR formation [22,27,28]. Specifically, a higher ratio of auxin to cytokinin, as well as a higher ratio of auxin to abscisic acid, can achieve a better rooting rate [19,39,41]. The present study indicated that the ratios of auxin to ABA as well as to tZR all showed an upward trend during the AR formation of *C. paliurus* soft cuttings (Figure 3b), consistent with the above-mentioned studies. It is worth noting that the IAA/tZR ratio decreased during the IE stage but increased in the following stages (Figure 3b). Thus, we speculate that a lower IAA/tZR ratio may be more conducive to the formation of root primordia in *C. paliurus* cuttings, whereas a higher IAA/tZR ratio would promote callus formation and root elongation in cuttings. Owing to the different roles that GA may play in the different stages of AR formation, the dynamics of the auxin/GA_1_ ratios varied from stage to stage but showed an overall downward tendency (Figure 3b), suggesting that a lower auxin/GA_1_ ratio should be beneficial for the formation of root primordia, callus formation, and root elongation in the *C. paliurus* cuttings.

### 3.3. Regulatory Mechanisms of Endogenous Hormones during the AR Formation Process

The dynamics of the DEGs related to important transcription factors of the hormone signaling pathway can partly explain the dynamics of hormone levels, while transcriptome analysis enables us to further understand the regulation mechanism of endogenous hormones during the AR formation process [47]. In the present study, most genes related to auxin influx carriers (AUXI) were down-regulated after cuttage due to the IAA loss in the cuttings (Figure 5). The possible reason is that the specific AUX/IAA proteins recruit TOPLESS (TPL) to exert their repressive function on specific auxin response factors (ARFs) under a low IAA level, allowing for the ubiquitination and subsequent proteasomal degradation of AUX/IAA proteins so that the ARFs are released from repression [15,23]. The release of ARFs activates the downstream transcription of GRETCHEN HAGEN3s (GH3) and SMALL AUXIN UP RNAs (SAUR) [47,48,49]. Similarly, the DEGs assigned to the key transcription factors of CK, GA, and ABA signals can also reflect the dynamics of the corresponding hormones (Figure 5).

**Figure 7:**
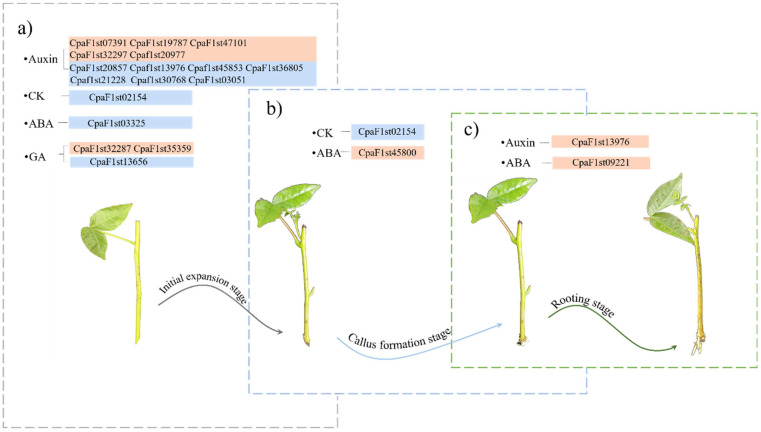
Key genes involved in hormone regulation at different AR formation stages of *C. paliurus* cuttings. (**a**) The expression of the key genes involved in hormonal regulation during the initial expansion stage. (**b**) The expression of the key genes involved in hormonal regulation during the callus formation stage. (**c**) The expression of the key genes involved in hormonal regulation during the rooting stage. CK, GA, and ABA represent cytokinin, gibberellin, and abscisic acid, respectively. Orange blocks indicate the up-regulated genes, while the blue ones show the down-regulated genes.

Based on the WGCNA analyses (Figure 6 and Table S4), the genes that are highly involved in hormonal regulation at different AR formation stages were screened in *C. paliurus* soft cuttings (Figure 7). In the auxin signal transduction pathway, *LAX*s (CpaF1st45853 and CpaF1st13976), *AUX*s (CpaF1st47101, CpaF1st20857, and CpaF1st36805), *IAA*s (CpaF1st30768, CpaF1st20977, and CpaF1st21228), *ARF*s (CpaF1st07391 and CpaF1st19787), *GH3.6* (CpaF1st32297), and *SAUR*s (CpaF1st16217, CpaF1st3051, and CpaF1st31602) are considered to play essential roles in regulating the IAA concentration at the IE stage during the AR formation of *C. paliurus* cuttings. However, *LAX2* (CpaF1st13976) was up-regulated at the RT stage, indicating that it might be one of the most important genes regulating the improvement in the IAA level so as to promote the AR formation of *C. paliurus* soft cuttings.

**Figure 8:**
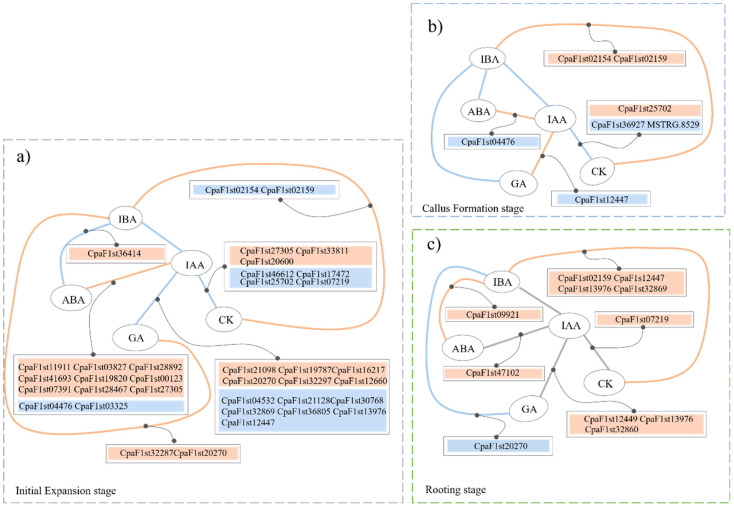
Auxin cross-regulatory interactions between auxin and various hormones during the AR formation of *C. paliurus* cuttings: (**a**), (**b**), and (**c**) show the hormone cross-regulatory interactions at the stages of initial expansion, callus formation, and rooting, respectively. CK, GA, and ABA represent cytokinin, gibberellin, and abscisic acid, respectively. The orange line indicates a positive correlation between hormones, the blue line indicates a negative correlation, and the gray line indicates no significant correlation between the hormones. Orange blocks indicate up-regulated genes, while the blue ones show the down-regulated genes.

Figure 7 also suggests that the negative response regulator A-ARR family gene *AAR4* (CpaF1st02154) might be the crucial gene in regulating the dynamics of cytokinin level, promoting root primordial induction and callus formation, whereas the GA receptor GID1-related gene *GID1B* (CpaF1st32287) and the GA signaling repressor DELLA-related gene *GAIPB* (CpaF1st13656) might be the important genes resulting in the increase of GA level in initial expansion. In terms of the regulation of ABA, the SNF1-related protein kinase2 (SnK2A)-related gene *SnK2A* (CpaF1st03325) may be the essential gene regulating the decrease in ABA level in the IE stage, and the ABA receptor-related gene *PYL4* (CpaF1st09921 and CpaF1st45800) possibly regulates the ABA level during the CF and RT stages, respectively. Moreover, the crosstalk varied at different AR formation stages of *C. paliurus* soft cuttings (Figure 8 and Table S6). For instance, a negative correlation between IAA and IBA in the IE and CF stages was detected, while IBA had a significant negative correlation with ABA in the IE stage but a positive correlation with tZR and GA_1_. However, there was a significant correlation between IBA and other hormones in the RT stage, while no significant correlations between IAA and other hormones were observed.

AUX/IAA proteins have been confirmed to be the cross-nodes to other plant hormones, such as cytokinin, abscisic acid, and gibberellin, by forming diverse dimers with auxin response factors (ARFs) [23,50]. Some studies have reported that *AUX/IAA*s targeting *AARR*s can mediate the regulation of cytokinin and stimulate root development in Arabidopsis [27,41], whereas the *AHP* family, such as *AHP6*, is another cross-node for regulating cytokinin under auxin signaling [27,51,52]. Our study showed that *IAA11* (CpaF1st36927), *AHP1* (CpaF1st33811), and *ARR9* (CpaF1st25702, CpaF1st07219, CpaF1st25702, and CpaF1st17472) were highly involved in the interaction between IAA and CK at different AR formation stages of *C. paliurus* soft cuttings (Figure 8), suggesting that *AHP*s, *AARR*s, and certain downstream ARF/IAA complexes would mediate the interaction between IAA and CK at the IE stage, whereas *AARR*s could be the key cross-node of the interaction throughout the AR formations. 

In terms of the interaction between IAA and ABA, some recent studies indicated that *PYL*s could interact with *MYB*s to enhance the expression levels of ARFs and with downstream *LBD*s to participate in the positive regulation of auxin biosynthesis in root growth [44,53], while *SAUR19* affected the root development of Arabidopsis roots [54,55]. In our study, *PYL*s (CpaF1st28892, CpaF1st00123, CpaF1st41693, etc.), *ARF*s (CpaF1st07319, CpaF1st28464, and CpaF1st47102), *IAA14* (CpaF1st04476 and CpaF1st047102), and *SAUR*s (CpaF1st11455 and CpaF1st04797) were demonstrated to be highly correlated to the interactions between auxin and ABA during the AR formation of *C. paliurus* soft cuttings. Thus, we guess that *PYL*s with the downstream *SAUR*s and ARF/IAA complexes may mediate the cross-talks between auxin and ABA to regulate hormone homeostasis and promote AR formation in *C. paliurus* soft cuttings.

## 4. Materials and Methods

### 4.1. Plant Materials

All healthy softwood shoots of *C. paliurus* were collected from the re-sprouting shoots of stumped seedlings (Jinghongshan 27#, a half-sib family) with a two-year root system on 20 May 2021, grown in the greenhouse of the Baima Teaching Experimental Base of Nanjing Forestry University (119°18′ E; 31°61′ N). The collected shoots were cut into cuttings for cutting propagation using the stainless steel pruning shears in the greenhouse. The prepared cuttings were 12–15 cm in length with 1 bud and 2 leaflets.

### 4.2. Experimental Design and Treatment Description

Three rooting agents (powders) were used as the materials to form different hormone-based formulas in the experiment. These rooting agents were ABT rooting powder #1 (ABT-1, Beijing, China), I-ROOT #3 (IR3, Winnipeg, MB, Canada) and STIM-ROOT No. 3 (SR3, Ottawa, ON, Canada). The main active ingredients in IR3 are two forms of IBA, free acid, and potassium salt, while IBA is the key component in SR3. However, the main active ingredients in ABT-1 are naphthylacetic acid (NAA) and IBA. According to the mass proportions of the rooting agents (SR3, IR3, and ABT-1), ten treatments were designed in total, namely H1 (SR3 only), H2 (IR3 only), H3 (SR3:IR3 = 1:1), H4 (SR3:IR3 = 1:4), H5 (SR3:IR3 = 4:1), H6 (SR3:IR3 = 2:3), H7 (SR3:IR3 = 3:2), H8 (ABT-1:SR3 = 1:1), H9 (ABT-1:IR3 = 1:1), and ck (ABT-1 only). 

This experiment was carried out in a greenhouse of the Baima Teaching Experimental Base, with the air temperature ranging from 20 to 30 °C and the relative humidity being 70–85% during the experimental period. After the disinfection of 2 min with 0.5% carbendazim, the bottom incision of the prepared cuttings was dipped with powders of the different formulas and then inserted into a moist medium of 1 perlite:3 peat:1 river sand (*v*/*v*) that was sterilized with 3000 mg L^−1^ chlorothalonil. Moreover, the cuttings were covered with a thin plastic sheet to retain the temperature and humidity of the air and medium after insertion.

A randomized complete block design was employed with six replications. There were 20 cuttings for each replicate, and a total of 1200 cuttings were used in this experiment. However, rooting observation and sampling were only performed in three replicates for each treatment, whereas another three replicates (a total of 60 cuttings for each treatment) remained undisturbed to check the final rooting parameters at the end of the experiment.

### 4.3. Rooting Process Observation and Sampling

**Figure 9:**
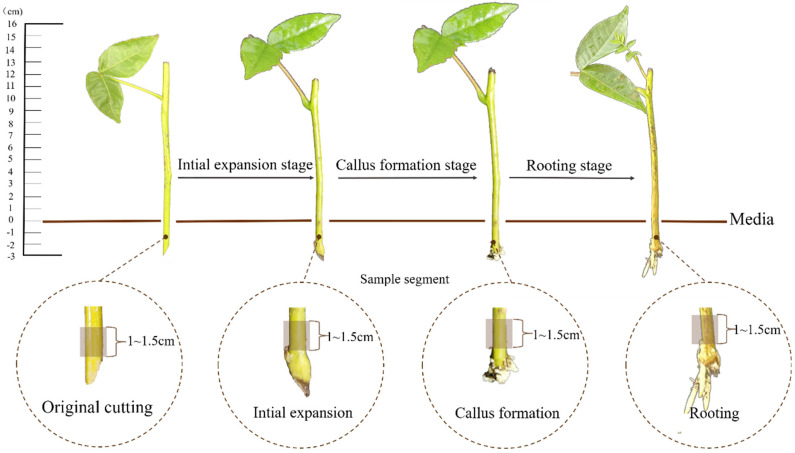
Division of three stages in the rooting process and segment sampling of *C. paliurus* cuttings for endogenous hormone content and transcriptome analyses.

Based on the results from our pre-experiment, we sampled the cuttings to check for the rooting process on days 15, 20, and 30 after cuttage. In each sampling time, 15 cuttings were sampled for each treatment (e.g., 5 cuttings for each replicate) to record the rooting status. The rooting process was divided into three stages in this study (Figure 9), i.e., stage 1 (initial expansion, IE), stage 2 (callus formation, CF), and stage 3 (rooting, RT). In total, 150 cuttings in each sampling time were collected and immediately frozen in liquid nitrogen and stored at −80 °C for further phytohormone measurement and RNA extraction. The final rooting parameters, such as the rooting rate, root number, and root length, were recorded and measured 60 days after treatment. 

According to the rooting status records and final rooting parameters after 60 days of treatment, only the samples collected from the H3 (SR3:IR3 = 1:1) treatment with the highest rooting rate were selected to conduct endogenous hormone and transcriptome analyses. To understand the temporal dynamics of endogenous hormones and gene expression during AR development, the basal cortex (1–1.5 cm) of the cuttings was sampled from the original cutting (OC), as well as the cuttings in the IE, CF, and RT stages (Figure 9).

### 4.4. Assessment of the Rooting Rate and Rooting Quality Indexes

The rooting rate and rooting quality indexes were determined 60 days after cuttage in the undisturbed treatments mentioned above. The data were collected for the number of rooted cuttings, number of roots per cutting, individual root length (cm), and the number of lateral roots per cutting during the assessment. The root quality index (RQI) per replicate for each treatment was evaluated according to the following formula:

RQI = (Rooting rate × root number per rooted cutting × mean root length)/100

### 4.5. Measurement of Endogenous Hormones

The IAA, IBA, trans-Zeatin-riboside (tZR, a kind of CK), GA_1_ (a kind of GA), and ABA portions were extracted and measured, as described previously by Pan et al. [56], with modifications. Briefly, about 1 g of fresh phloem tissue from *C. paliurus* soft cuttings, sampled according to the description in Figure 9, was ground into a fine powder in liquid nitrogen, and then the phytohormone was extracted twice with acetonitrile and purified with a Poroshell 120 SB-C18 column. The quantification of phytohormones was determined by LC-MS (Agilent 1290 Infinity, Agilent Technologies, Palo Alto, CA, USA) with a SCIEX 6500Qtrap (Framingham, MA, USA) on 20 March 2022. These experiments were repeated three times with similar results and carried out by RUIYUAN Biotechnology Co., Ltd. (Nanjing, China; www.bestofbest.top).

### 4.6. RNA Extraction and Transcriptomics Analysis

The extraction of total RNA was based on the company’s instructions, and the RNA integrity assessment was carried out using the Agilent 2100 Bioanalyzer (Agilent Technologies, Palo Alto, CA, USA). The cDNA library was constructed for each sample using high-throughput sequencing (Illumina HiSeqTM 4000) by Gene Denovo Biotechnology Co. (Guangzhou, China), and a total of 12 libraries were constructed, e.g., including samples collected at OC, IE, CF, and RT statuses (three biological replicates for each status). The raw reads from the transcriptome sequencing were filtered using Fastp (Version 0.18.0) to obtain high-quality clean reads [57,58]. The HISAT2 tool was used to map the cleaned and filtered reads for further analysis and annotation. Transcript expression abundant and gene expression levels were calculated via the method of fragments per kilobase per transcript per million mapped reads (FPKM) [6]. Genes with |log2FC| > 1 and FDR < 0.05 were defined as differentially expressed genes (DEGs). The DESeq2 R package was used to analyze the DEGs between two groups. All the DEGs were annotated to public databases, including the Gene Ontology (GO) (http://www.geneontology.org/) and Kyoto Encyclopedia of Genes and Genomes (KEGG) (https://www.genome.jp/) databases.

To identify the genes that are highly correlated with the dynamics of phytohormone concentration and involved in hormone signal transduction during the AR formation of *C. paliurus* soft cuttings, a weighted gene co-expression network analysis (WGCNA) was performed using the OmicShare tools (https://www.omicsmart.com/) based on the dynamics of the phytohormone concentration and all DEGs identified in all collected samples. The adjacency matrix between different genes was constructed with a threshold power of 8, while a dynamic tree cut procedure (merge cut height = 0.9; min module size = 50) was used to screen similar modules in the hierarchical tree. The module eigengene was defined as the first principal component of a given module and then used to represent the expression profile of module genes in each sample. Based on the Pearson correlation analysis between module feature values and the phytohormone concentration dynamics, performed using the R package ggplot2, we identified the 5 modules that are most relevant to the phytohormone concentration changes. After that, the genes related to the trait in each module were screened in terms of the gene significance (GS) values between each gene and each hormone concentration. Finally, by means of the GS value and transcriptome DEG analysis, we dug out the key genes involved in hormone regulation with GS value > 0.7 for each AR formation stage.

### 4.7. Quantitative Real-Time (qRT-PCR) Validation

To validate the RNA-seq data, nine genes were randomly chosen for validation using qRT-PCR. According to the manufacturer’s instructions, total RNA was extracted using a Trizol reagent kit (Invitrogen, Carlsbad, CA, USA) from the samples of *C. paliurus* cuttings in the OC, IE, CF, and RT stages. A NanoDrop 2000 Spectrophotometer (Thermo Scientific, Waltham, MA, USA) and 1% agarose gel electrophoresis were used to assess the total RNA concentration and quality. The cDNA was obtained using MonScript RTIII All-in-One Mix with dsDNase kits (Monad Biotech Co., Ltd., Suzhou, China). SYBR Green Realtime PCR Master Mix Kit (Toyobo Co., Ltd., Osaka, Japan) and an Applied BiosystemsTM 7500 Real-Time PCR System (Monad, China) were used for qRT-PCR analysis. Each sample included three biological and technical replicates. The National Center for Biotechnology Information (NCBI) online tool (http://www.ncbi.nlm.nih.gov/) was used to design the primers, and the primer sequences for qRT-PCR are shown in Table S7. The relative expression levels of the target genes were quantified using the 2^−ΔΔCt^ formula [59].

### 4.8. Statistical Analysis

All the statistical analyses were conducted using GraphPad Prism version 9.0 for Windows (GraphPad software, San Diego, CA, USA). Differences between samples were determined through the one-way analysis of variance (ANOVA), and significant differences were calculated using the least significant difference (LSD) test at *p* < 0.05.

## 5. Conclusions

The application of different hormone-based formulas significantly affects the rooting rate, root parameters, and root quality index of *C. paliurus* softwood cuttings (*p* < 0.05). The best rooting rate and root quality index were achieved in the H3 formula (SR3:IR3 = 1:1), reaching 93% and 89, respectively. During the AR formation of *C. paliurus* softwood cuttings, the dynamics for each phytohormone and their ratio varied greatly, suggesting that there exists an optimum trade-off among the phytohormones in each AR formation stage and that this trade-off would be stage-specific. The transcriptome analyses show that 19 genes with vital functions (such as *LAX*s, *IAA*s, etc.) are highly associated with hormone regulation at different AR formation stages in *C. paliurus* softwood cuttings. Our findings would not only optimize a hormone-based formula for improving cutting rooting of *C. paliuru*s but also provide an insight into the hormonal regulatory network of this hard-to-root tree species. However, the molecular mechanism of hormonal interactions during the AR formation of *C. paliurus* soft cuttings needs to be further studied.

## Data Availability

The data presented in this study are available in the article and Supplementary Materials.

## Supplementary Materials

The following supporting information can be downloaded at: https://www.mdpi.com/article/10.3390/ijms25021343/s1.

## References

[1] Zhang S., He J., Li J., He H., He Y., Wang X., Shu H., Zhang J., Xu D., Zou K. (2023). Triterpenoid compounds from *Cyclocarya paliurus*: A review of their phytochemistry, quality control, pharmacology, and structure–activity relationship. Am. J. Chin. Med..

[2] Manchester S.R., Chen Z., Lu A., Uemura K. (2009). Eastern Asian endemic seed plant genera and their paleogeographic history throughout the Northern Hemisphere. J. Syst. Evol..

[3] Qin J., Yue X., Fang S., Qian M., Zhou S., Shang X., Yang W. (2021). Responses of nitrogen metabolism, photosynthetic parameter and growth to nitrogen fertilization in *Cyalocarya paliurus*. For. Ecol..

[4] Zhou M., Chen P., Lin Y., Fang S., Shang X. (2019). A comprehensive assessment of bioactive metabolites, antioxidant and antiproliferative activities of *Cyclocarya paliurus* (Batal.) Iljinskaja Leaves. Forests.

[5] Fang S., Sun D., Shang X., Fu X., Yang W. (2020). Variation in radial growth and wood density of *Cyclocarya paliurus* across its natural distribution. New For..

[6] Zhang L., Zhang Z., Fang S., Liu Y., Shang X. (2021). Integrative analysis of metabolome and transcriptome reveals molecular regulatory mechanism of flavonoid biosynthesis in *Cyclocarya paliurus* under salt stress. Ind. Crops Prod..

[7] Zhou M., Chen P., Shang X., Yang W., Fang S. (2021). Genotype–environment interactions for tree growth and leaf phytochemical content of *Cyclocarya paliurus* (Batal.) Iljinskaja. Forests.

[8] Kakar M., Naveed M., Saeed M., Zhao S., Rasheed M., Firdoos S., Manzoor R., Deng Y., Dai R. (2020). A review on structure, extraction, and biological activities of polysaccharides isolated from *Cyclocarya paliurus* (Batalin) Iljinskaja. Int. J. Biol. Macromol..

[9] Lan L., Xu Z., Sun C., Fang S. (2022). Evaluation on germplasm resources of *Cyclocarya paliurus* and its oriented selection of superior families and trees. For. Res..

[10] Thomas A., Brauer D., Sauer T., Coggeshall M.V., Ellersieck M. (2008). Cultivar influences early rootstock and scion survival of grafted black walnut. J. Amer. Pomolog. Soc..

[11] Stevens M.E., Pijut P.M. (2017). Origin of adventitious roots in black walnut (*Juglans nigra*) softwood cuttings rooted under optimized conditions in a fog chamber. New For. Tree Physiol..

[12] Li N., Zhu P., Feng C., Wen M., Fang S., Shang X. (2021). Variation in physiological characteristics of rootstock-scion and its relationship to graft compatibility during the grafting union process of *Cyclocarya paliurus*. J. Nanjing For. Univ..

[13] Yang W., Zhuang J., Ding S., Zhang M., Tian Y., Wan S., Fang S. (2022). Study on cutting cultivation technology and rooting mechanism of *Cyclocarya paliurus*. Ecol. Chem. Eng. S.

[14] Wan S.Y., Tian Y., Yang W., Fang S. (2023). Effects of different hormone formulas on nutrient substance, enzyme activity and rooting in hardwood cutting of *Cyclocarya paliurus*. Non-Wood. For. Res..

[15] Druege U., Hilo A., Pérez-Pérez J.M., Klopotek Y., Acosta M., Shahinnia F., Zerche S., Franken P., Hajirezaei M.R. (2019). Molecular and physiological control of adventitious rooting in cuttings: Phytohormone action meets resource allocation. Ann. Bot..

[16] Villacorta-Martín C., Belén Sánchez-García A., Villanova J., Cano A., Van De Rhee M., De Haan J., Acosta M., Passarinho P., Manuel Pérez-Pérez J. (2015). Gene expression profiling during adventitious root formation in carnation stem cuttings. BMC Genom..

[17] Druege U., Franken P., Hajirezaei M.R. (2016). Plant hormone homeostasis, signaling, and function during adventitious root formation in cuttings. Front. Plant Sci..

[18] Ren H., Hu H., Luo X., Zhang C., Li X., Li P., Li W., Khawar A., Sun X., Ren Z. (2019). Dynamic changes of phytohormone signaling in the base of *Taxus media* stem cuttings during adventitious root formation. Sci. Hortic..

[19] Wang Y., Pang D., Ruan L., Liang J., Zhang Q., Qian Y., Zhang Y., Bai P., Wu L., Cheng H. (2022). Integrated transcriptome and hormonal analysis of naphthalene acetic acid-induced adventitious root formation of tea cuttings (*Camellia sinensis*). BMC Plant Biol..

[20] Bustillo-Avendaño E., Ibáñez S., Sanz O., Barros J.A.S., Gude I., Perianez-Rodriguez J., Micol J.L., del Pozo J.C., Moreno-Risueno M.A., Pérez-Pérez J.M. (2018). Regulation of hormonal control, cell reprogramming, and patterning during de novo root organogenesis. Plant Physiol..

[21] Pacurar D.I., Perrone I., Bellini C. (2014). Auxin is a central player in the hormone cross-talks that control adventitious rooting. Physiol. Plant..

[22] da Costa C.T., de Almeida M.R., Ruedell C.M., Schwambach J., Maraschin F.S., Fett-Neto A.G. (2013). When stress and development go hand in hand: Main hormonal controls of adventitious rooting in cuttings. Front. Plant Sci..

[23] Yu Z., Zhang F., Friml J., Ding Z. (2022). Auxin signaling: Research advances over the past 30 years. J. Integr. Plant Biol..

[24] Ahkami A.H., Melzer M., Ghaffari M.R., Pollmann S., Ghorbani Javid M., Shahinnia F., Hajirezaei M.R., Druege U. (2013). Distribution of indole-3-acetic acid in *Petunia hybrida* shoot tip cuttings and relationship between auxin transport, carbohydrate metabolism and adventitious root formation. Planta.

[25] Gutierrez L., Mongelard G., Floková K., Pǎcurar D.I., Novák O., Staswick P., Kowalczyk M., Păcurar M., Demailly H., Geiss G. (2012). Auxin controls Arabidopsis adventitious root initiation by regulating jasmonic acid homeostasis. Plant Cell.

[26] Wang L., Zhang Y., Liu X. (2022). Study on young branch cutting seedling technology of *Cyclocarya paliurus*. Prot. For. Sci. Technol..

[27] Liu J., Moore S., Chen C., Lindsey K. (2017). Crosstalk complexities between auxin, cytokinin, and ethylene in *Arabidopsis* root development: From experiments to systems modeling, and back again. Mol. Plant.

[28] Saito T., Opio P., Wang S., Ohkawa K., Kondo S., Maejima T., Ohara H. (2019). Association of auxin, cytokinin, abscisic acid, and plant peptide response genes during adventitious root formation in Marubakaido apple rootstock (*Malus prunifolia* Borkh. var. ringo Asami). Acta. Physiol. Plant.

[29] Lakehal A., Bellini C. (2019). Control of adventitious root formation: Insights into synergistic and antagonistic hormonal interactions. Physiol. Plant..

[30] Ptošková K., Szecówka M., Jaworek P., Tarkowská D., Petřík I., Pavlović I., Novák O., Thomas S.G., Phillips A.L., Hedden P. (2022). Changes in the concentrations and transcripts for gibberellins and other hormones in a growing leaf and roots of wheat seedlings in response to water restriction. BMC Plant Biol..

[31] Hernández-García J., Briones-Moreno A., Blázquez M.A. (2021). Origin and evolution of gibberellin signaling and metabolism in plants. Semin. Cell. Dev. Biol..

[32] Jing W., Zhang S., Fan Y., Deng Y., Wang C., Lu J., Sun X., Ma N., Shahid M.O., Li Y. (2020). Molecular Evidences for the Interactions of Auxin, Gibberellin, and Cytokinin in Bent Peduncle Phenomenon in Rose (*Rosa* sp.). Int. J. Mol. Sci..

[33] Qu Y., Kong W., Wang Q., Fu X. (2021). Genome-wide identification MIKC-Type MADS-Box gene family and their roles during development of floral buds in wheel wingnut (*Cyclocarya paliurus*). Int. J. Mol. Sci..

[34] Hu J., Israeli A., Ori N., Sun T.P. (2018). The interaction between DELLA and ARF/IAA mediates crosstalk between gibberellin and auxin signaling to control fruit initiation in tomato. Plant Cell.

[35] Zhang J., Guo Z., Zhang R., Li Y., Cao L., Liang Y., Huang L. (2015). Auxin type, auxin concentration, and air and substrate temperature difference play key roles in the rooting of juvenile hardwood pecan cuttings. Hort. Technol..

[36] Guo C., Shangguan X., Jiang Y., Yang W., Zhang J.W. (2008). A study on the effects of growth regulators on cutting techniques of *Cyclocarya paliurus*. Acta. Agri. Univ. Jiangxiensis.

[37] Zhang G. (2016). A study on soft cutting propagation technique of *Cyclocarya paliurus* in the southern region of Fujian province. Anhui Agri. Sci. Bull..

[38] Du W., Cheng J. (2014). Morphological and anatomical observation on cortex rooting process of mulberry (*Morus* L.) greenwood cuttings. Sci. Seri..

[39] Liu G., Zhao J., Liao T., Wang Y., Guo L., Yao Y., Cao J. (2021). Histological dissection of cutting-inducible adventitious rooting in *Platycladus orientalis* reveals developmental endogenous hormonal homeostasis. Ind. Crops. Prod..

[40] Zhang J., Shi J., Xia G., Huang Y., Huang J., Wang Z. (2014). Research of lignified cuttings and rooting mechanism of *Carya illinoensis*. J. Anhui Agric. Univ..

[41] Otiende M.A., Fricke K., Nyabundi J.O., Ngamau K., Hajirezaei M.R., Druege U. (2021). Involvement of the auxin-cytokinin homeostasis in adventitious root formation of rose cuttings as affected by their nodal position in the stock plant. Planta.

[42] Zhang F., Wang O. (2019). Research status of rooting mechanism of peach hardwood cutting. Plant Physiol. J..

[43] Quan J., Ni R., Wang Y., Sun J., Ma M., Bi H. (2022). Effects of different growth regulators on the rooting of *Catalpa bignonioides* softwood cuttings. Life.

[44] Zhang J., Zhou T., Zhang C., Zheng W., Li J., Jiang W., Xiao C., Wei D., Yang C., Xu R. (2021). Gibberellin disturbs the balance of endogenesis hormones and inhibits adventitious root development of *Pseudostellaria heterophylla* through regulating gene expression related to hormone synthesis. Saudi. J. Bio. Sci..

[45] Busov V., Meilan R., Pearce D.W., Rood S.B., Ma C., Tschaplinski T.J., Strauss S.H. (2006). Transgenic modification of gai or *rgl1* causes dwarfing and alters gibberellins, root growth, and metabolite profiles in *Populus*. Planta.

[46] Steffens B., Wang J., Sauter M. (2006). Interactions between ethylene, gibberellin and abscisic acid regulate emergence and growth rate of adventitious roots in deepwater rice. Planta.

[47] Chen R., Zhao D., Huang X. (2022). Transcriptome analysis of easy- and hard-to-root tea plants uncovers roles for *CsGH3.2* and *CsGH3.3* in adventitious root formation. Plant Cell Tissue Organ Cult..

[48] Yuan H., Zhao K., Lei H., Shen X., Liu Y., Liao X., Li T. (2013). Genome-wide analysis of the *GH3* family in apple (*Malus* × *domestica*). BMC Genom..

[49] Hu Y., Omary M., Hu Y., Doron O., Hoermayer L., Chen Q., Megides O., Chekli O., Ding Z., Friml J. (2021). Cell kinetics of auxin transport and activity in Arabidopsis root growth and skewing. Nat. Commun..

[50] Luo J., Zhou J., Zhang J. (2018). Aux/IAA gene family in plants: Molecular structure, regulation, and function, *Int*. J. Mol. Sci..

[51] Chandler J.W., Werr W. (2015). Cytokinin-auxin crosstalk in cell type specification. Trends Plant Sci..

[52] Li T., Kang X., Lei W., Yao X., Zou L., Zhang D., Lin H. (2020). SHY2 as a node in the regulation of root meristem development by auxin, brassinosteroids, and cytokinin. J. Integr. Plant Biol..

[53] Li J., Xu P., Zhang B., Song Y., Wen S., Bai Y., Ji L., Lai Y., He G., Zhang D. (2023). Paclobutrazol promotes root development of difficult-to-root plants by coordinating auxin and abscisic acid signaling pathways in *Phoebe bournei*. Int. J. Mol. Sci..

[54] Wong J., Spartz A.K., Park M., Du M., Gray W. (2019). Mutation of a conserved motif of PP2C.D phosphatases confers SAUR immunity and constitutive activity. Plant Physiol..

[55] Emenecker R.J., Strader L.C. (2008). Auxin-Abscisic acid interactions in plant growth and development. Biomolecules.

[56] Pan X., Welti R., Wang X. (2010). Quantitative analysis of major plant hormones in crude plant extracts by high-performance liquid chromatography-mass spectrometry. Nat. Protoc..

[57] Chen S., Zhou Y., Chen Y., Gu J. (2018). Fastp: An ultra-fast all-in-one FASTQ preprocessor. Bioinformatics.

[58] Li Y., Zhang J., Wang S., Zhang H., Liu Y., Yang M. (2023). Integrative transcriptomic and metabolomic analyses reveal the flavonoid biosynthesis of *Pyrus hopeiensis* flowers under cold stress. Hortic. Plant J..

[59] Livak K.J., Schmittgen T.D. (2001). Analysis of relative gene expression data using real time quantitative PCR and the 2^−ΔΔCT^. Methods.

