# Supplementary material for: Insights into the Hormone-Regulating Mechanism of Adventitious Root Formation in Softwood Cuttings of Cyclocarya paliurus and Optimization of the Hormone-Based Formula for Promoting Rooting
Source: Int J Mol Sci. 2024 Jan 22;25(2):1343. doi: 10.3390/ijms25021343 (PMC10816064; doi:10.3390/ijms25021343)
Supplement: Supplementary file 1 [file ijms-25-01343-s001.zip › ijms-2799269-supplementary.pdf]

Supplementary Materials

Supplementary Tables:

Table S1. Data quality control of each sample

Sample	RawData(bp)	CleanData(bp)	AF_Q20(%)	AF_Q30(%)
U1-1	9490722600	9418260696	9197835879 (97.66%)	8796407176 (93.40%)
U1-2	7557417900	7495097298	7335059648 (97.86%)	7038460496 (93.91%)
U1-3	8235694500	8175443475	7997345896 (97.82%)	7670651078 (93.83%)
U2-1	6369126600	6314814509	6169616813 (97.70%)	5907214227 (93.55%)
U2-2	6376837500	6308766172	6163290061 (97.69%)	5902739296 (93.56%)
U2-3	7755867600	7676697245	7503392629 (97.74%)	7189874345 (93.66%)
U3-1	7072183800	7014121261	6853234916 (97.71%)	6561645323 (93.55%)
U3-2	6400034700	6340363197	6205864456 (97.88%)	5958509348 (93.98%)
U3-3	5993775900	5941506824	5807306117 (97.74%)	5564318136 (93.65%)
U4-1	6920610900	6854434459	6703931457 (97.80%)	6430253779 (93.81%)
U4-2	7794695400	7713384952	7528826847 (97.61%)	7198727547 (93.33%)
U4-3	7549488600	7473771094	7290677261 (97.55%)	6963362886 (93.17%)
D1-1	7670463600	7610098370	7434553718 (97.69%)	7114209567 (93.48%)
D1-2	5866717500	5822746868	5684758740 (97.63%)	5434398995 (93.33%)
D1-3	6450338700	6400478841	6254625660 (97.72%)	5983949434 (93.49%)
D2-1	5544637200	5495810405	5370926819 (97.73%)	5143465060 (93.59%)
D2-2	6445602600	6394662952	6237115813 (97.54%)	5957633440 (93.17%)
D2-3	6768116700	6711472620	6551332713 (97.61%)	6261938095 (93.30%)
D3-1	6586533000	6542253067	6397743406 (97.79%)	6131448013 (93.72%)
D3-2	6627561000	6580012022	6431709914 (97.75%)	6161301103 (93.64%)
D3-3	5664131400	5624376803	5487539031 (97.57%)	5243303379 (93.22%)
D4-1	6147993900	6096540677	5949499232 (97.59%)	5689610392 (93.33%)
D4-2	7663922700	7605702496	7426294152 (97.64%)	7106420480 (93.44%)
D4-3	6219270000	6163713409	6017681710 (97.63%)	5756940268 (93.40%)

Table S2. Mapping rate of each sample

Sample	Total reads	Unmapped(%)	Total_Mapped(%)
U1-1	62994504	4668823 (7.41%)	58325681 (92.59%)
U1-2	50194716	3600793 (7.17%)	46593923 (92.83%)
U1-3	54698204	4008810 (7.33%)	50689394 (92.67%)
U2-1	42257630	5012746 (11.86%)	37244884 (88.14%)
U2-2	42248994	5047782 (11.95%)	37201212 (88.05%)
U2-3	51457108	6140500 (11.93%)	45316608 (88.07%)
U3-1	46921356	3917887 (8.35%)	43003469 (91.65%)
U3-2	42460646	3519019 (8.29%)	38941627 (91.71%)
U3-3	39782988	3335641 (8.38%)	36447347 (91.62%)
U4-1	45946242	3958144 (8.61%)	41988098 (91.39%)
U4-2	51721426	4719352 (9.12%)	47002074 (90.88%)
U4-3	50085242	4466399 (8.92%)	45618843 (91.08%)
D1-1	50930700	3837443 (7.53%)	47093257 (92.47%)
D1-2	38984134	2920225 (7.49%)	36063909 (92.51%)
D1-3	42863422	3163663 (7.38%)	39699759 (92.62%)
D2-1	36823850	4521449 (12.28%)	32302401 (87.72%)
D2-2	42790900	5338677 (12.48%)	37452223 (87.52%)
D2-3	44931074	5505363 (12.25%)	39425711 (87.75%)
D3-1	43760866	5239789 (11.97%)	38521077 (88.03%)
D3-2	44027482	4556967 (10.35%)	39470515 (89.65%)
D3-3	37616308	3937969 (10.47%)	33678339 (89.53%)
D4-1	40820084	4333364 (10.62%)	36486720 (89.38%)
D4-2	50902698	5253024 (10.32%)	45649674 (89.68%)
D4-3	41297782	4266957 (10.33%)	37030825 (89.67%)

Table S3. Module Trait Relation Result

Trait	Module name	ModuleTraitRelationResults (pvalue)
IAA	MM.brown	-0.916088613577993(1.958149e-04)
	MM.darkgrey	-0.876994533634533(8.611645e-04)
	MM.lightcyan	-0.856363924979661(1.560062e-03)
	MM.turquoise	0.745846361405391(1.325512e-02)
	MM.plum2	-0.712504636744523(2.076147e-02)
IBA	MM.salmon	0.980124801431881(6.665428e-07)
	MM.cyan	0.842842370025318(2.197731e-03)
	MM.bisque4	-0.82635472111522(3.207316e-03)
	MM.lightcyan	0.822290881826334(3.499985e-03)
	MM.brown4	0.806643377772691(4.807499e-03)
CK	MM.brown4	0.990115866460472(4.126406e-08)
	MM.plum1	0.832808308585645(2.779292e-03)
	MM.salmon	0.826315043338519(3.210085e-03)
	MM.lightcyan	0.767469395349517(9.556031e-03)
	MM.bisque4	-0.753221704850762(1.189744e-02)
GA	MM.darkmagenta	0.932439663738384(8.396406e-05)
	MM.brown	0.895334064954696(4.619359e-04)
	MM.cyan	0.877657144959418(8.434598e-04)
	MM.turquoise	-0.875009331816558(9.158308e-04)
	MM.tan	0.78829705789873(6.746372e-03)
ABA	MM.orange	-0.968810898434766(3.986958e-06)
	MM.darkorange	-0.873060352246282(9.719176e-04)
	MM.darkgrey	-0.863954482017307(1.267631e-03)
	MM.ivory	-0.822580355644675(3.478528e-03)
	MM.bisque4	-0.788752239811132(6.692496e-03)

Table S4. Key genes involved in hormone signaling during the adventitious root formation of *C. paliurus* cuttings

Hormone	Gene ID	Symbol	GS	IEstage	CFstage	RTstage	Module
Auxin	CpaF1st07391	ARF3	0.83	1.11	-0.38	0.39	Darkgrey
	CpaF1st19787	ARF9	0.84	1.28	-0.01	-0.13	Brown
	CpaF1st47101	AUX22B	0.80	2.47	-0.06	0.19	Darkgray
	CpaF1st36805	AUX22D	0.81	-3.13	-0.76	0.74	Turquoise
	CpaF1st20857	AUX28	0.87	-1.72	-0.66	-0.31	Turquoise
	CpaF1st32297	GH3.6	0.74	1.72	0.16	-0.32	Turquoise
	CpaF1st20977	IAA11	0.91	2.58	-0.75	-0.01	Lightcyan
	CpaF1st21228	IAA27	0.84	-1.06	0.03	0.41	Darkgray
	CpaF1st30768	IAA29	0.80	-2.51	-0.70	0.84	Turquoise
	CpaF1st45853	LAX2	0.74	-1.54	-0.59	0.65	Lightcyan
	CpaF1st13976	LAX3	0.84	-3.26	-0.43	1.12	Darkgray
	CpaF1st03051	SAUR23	0.79	-2.13	-0.11	0.96	Turquoise
Cytokinin	CpaF1st02154	ARR4	0.86	-1.57	1.33	0.35	brown4
Gibberellin	CpaF1st13656	GAIPB	0.85	-1.16	-0.16	0.18	Turquoise
	CpaF1st32287	GID1B	0.88	1.96	-0.16	-0.87	Turquoise
	CpaF1st35359	PIF1	0.93	1.31	0.30	-0.33	Darkmagenta
Abscisic acid	CpaF1st09221	PYL4	0.72	-0.49	0.93	1.11	Orange
	CpaF1st45800	PYL4	0.75	0.59	1.13	0.59	Darkorange
	CpaF1st03325	SRK2A	0.83	-1.73	0.14	0.10	orange

Table S5. Key genes involved in hormonal interactions during the adventitious root formation of *C. paliurus* cuttings

Hormone		Gene ID	Symbol	Gs	IEstage	CFstage	RTstage	Module
Auxin-CK	IAA-CK	CpaF1st07219	ARR9	0.91	-2.46	0.80	1.04	brown
		CpaF1st17472	ARR9	0.95	-4.36	0.86	0.86	Turquoise
		CpaF1st20600	AHK2	0.97	2.31	-0.43	0.26	Lightcyan
		CpaF1st25702	ARR9	0.94	-3.83	1.12	0.19	Turquoise
		CpaF1st27305	RR23	0.94	1.88	-0.08	0.06	Turquoise
		CpaF1st33811	AHP1	0.95	3.96	-0.58	-0.34	Lightcyan
		CpaF1st36927	IAA11	0.92	0.29	-1.08	0.44	Darkgray
		CpaF1st46612	PHP5	0.96	-5.12	2.64	0.22	Turquoise
		MSTRG.8529	ARG7	0.82	-0.37	-1.39	0.14	Brown
	IBA-CK	CpaF1st02154	ARR4	0.96	-1.57	1.33	0.35	Salmon
		CpaF1st02159	ARR4	0.96	-2.19	1.38	1.04	Salmon
Auxin-GA	IAA-GA	CpaF1st04532	IAA8	0.95	-2.56	0.82	0.86	Darkgray
		CpaF1st12447	LAX5	0.97	-1.73	-1.27	1.58	Turquoise
		CpaF1st12660	SAUR-Like	0.93	3.78	0.08	-0.12	Turquoise
		CpaF1st13976	LAX3	0.93	-3.26	-0.43	1.12	Turquoise
		CpaF1st16217	SAUR36	0.97	2.95	-0.09	-0.73	Brown
		CpaF1st19787	ARF9	0.94	1.28	-0.01	-0.13	Turquoise
		CpaF1st21098	GAI1	0.97	1.25	-0.40	0.16	Lightcyan
		CpaF1st21228	IAA27	0.98	-1.06	0.03	0.41	Brown
		CpaF1st30768	IAA29	0.93	-2.51	-0.70	0.84	Turquoise
		CpaF1st32297	GH3.6	0.98	1.72	0.16	-0.32	Turquoise
		CpaF1st32869	IAA4	0.99	-2.39	-0.13	1.52	Brown
		CpaF1st36805	AUX22D	0.91	-3.13	-0.76	0.74	Turquoise

		CpaF1st13656	GAIPB	0.91	-1.16	-0.16	0.18	Turquoise
	IBA-GA	CpaF1st20270	GH3.6	0.92	4.10	-0.29	-1.13	Brown
		CpaF1st32287	GID1B	0.83	1.96	-0.16	-0.87	Turquoise
Auxin-ABA	IAA-ABA	CpaF1st00123	PYL3	0.92	1.26	-0.57	0.12	Lightcyan
		CpaF1st03325	SRK2A	0.94	-1.73	0.14	0.10	Turquoise
		CpaF1st03827	ABF4	0.91	1.49	0.06	-0.09	Turquoise
		CpaF1st04476	IAA14	0.81	-1.02	-2.34	0.08	Brown
		CpaF1st04797	SAUR71	0.89	2.10	0.21	0.60	Brown
		CpaF1st07391	ARF3	0.92	1.11	-0.38	0.39	Lightcyan
		CpaF1st11455	SAUR36	0.86	2.80	0.72	0.21	Brown
		CpaF1st11911	ABF3	0.89	1.92	0.06	-1.33	Darkgrey
		CpaF1st19820	PYL3	0.85	2.55	0.25	-0.21	Turquoise
		CpaF1st28467	ARF9	0.87	1.97	0.30	0.09	Brown
		CpaF1st28892	PYL1	0.89	3.16	0.17	-0.34	Turquoise
		CpaF1st41693	PYL9	0.99	1.79	-0.26	0.08	Lightcyan
		CpaF1st47102	IAA14	0.84	0.76	-0.14	2.16	Darkgrey
	IBA-ABA	CpaF1st09221	PYL4	0.87	-0.49	0.93	1.11	Bisgue4
		CpaF1st36414	PP2C06	0.91	3.82	-0.69	-1.06	Lightcyan

Table S6. Phytohormone correlation in *C. paliurus* soft cuttings at different stages in AR formation (n=12)

Stage	Hormone	IAA	IBA	ABA	tZR	GA_1
Initial expansion stage (IE)	IAA	1.00				
	IBA	-0.988**	1.00			
	ABA	0.993**	-0.987**	1.00		
	tZR	-0.963**	0.949**	-0.983**	1.00	
	GA_1	-0.979**	0.953**	-0.985**	0.992**	1.00
Callus formation stage (CF)	IAA	1				
	IBA	-0.934**	1			
	ABA	0.880*	-0.976**	1		
	tZR	-0.907*	0.979**	-0.967**	1	
	GA_1	0.858*	-0.882*	0.934**	-0.889*	1
Rooting stage (RT)	IAA	1				
	IBA	0.216	1			
	ABA	0.122	0.984**	1		
	tZR	-0.024	0.895*	0.911*	1	
	GA_1	-0.166	-0.975**	-0.990**	-0.918**	1

**. At 0.01 level (double tailed), the correlation is significant.

*. At 0.05 level (double tailed), the correlation is significant.

Table S7. Primers of sequences for qRT-PCR analysis

Gene ID	Forward primer (5'to 3')	Reverse primer (5'to 3')
CpaF1st45853	GGTATGGGTGCTGGTAGTCG	ATTTTTCGTGGCTGCGGTTC
CpaF1st21228	AAAGGTGGTCTTGTGGGTGG	GCGACGGCTTAGGTGACTG
CpaF1st20857	GCATCCTGCTAAGGCACAAG	CGCCAACGAGCATCCAGT
CpaF1st19787	TCAACCAGAAGTCGCACCAA	ATCGTCACCCACAAGCATCA
CpaF1st16217	CGAACGGGGCGAAAACA	GTAAATCACGGGCACCAGAA
CpaF1st32287	TCTTGACCGCAAAGTCCCTG	CAAGGCGGCGACAAAAAGTG
CpaF1st13656	ACGAGTCCTGTCCCTACCTG	CTGGCAACGAACCCTCTG
CpaF1st45800	GGTCCGTTACCACCCTTCAC	TACCAGGCGGCACATCAAC
CpaF1st03325	TGACGAGAATGTGGCGAGAG	CGATAGCGAGATGGGTAGGAGT
CpaF1st03194	GCATTCGTTGCGTTCTTCTCC	CAGCACCCCTGACTTCCTTG

Supplementary Figures:

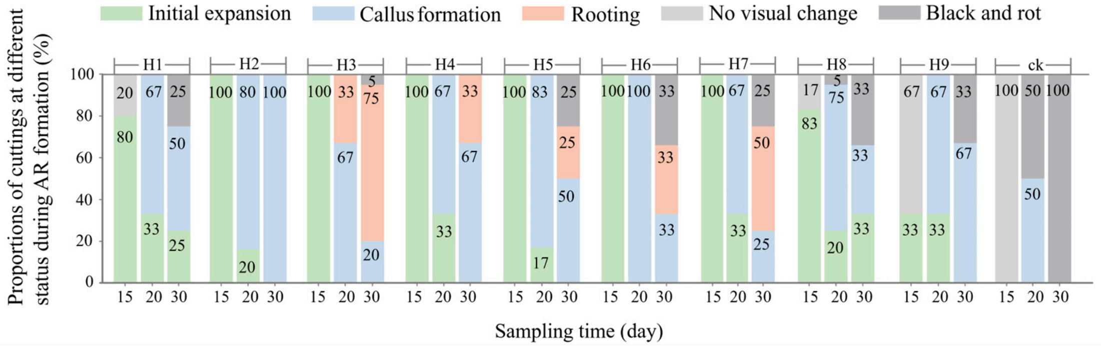

Figure S1. Differences in rooting process of *C. paliurus* soft cuttings under different hormone formulas

The numbers reveal the proportion of *C. paliurus* soft cuttings at different status during the adventitious root formation at different sampling times (%). Blocks with different colors represent the different status of *C. paliurus* soft cuttings. H1, H2, H3, H4, H5, H6, H7, H8, and H9 represent different hormone formula treatments respectively, while ck means a control group of *C. paliurus* soft cuttings treated by ABT-1 only.

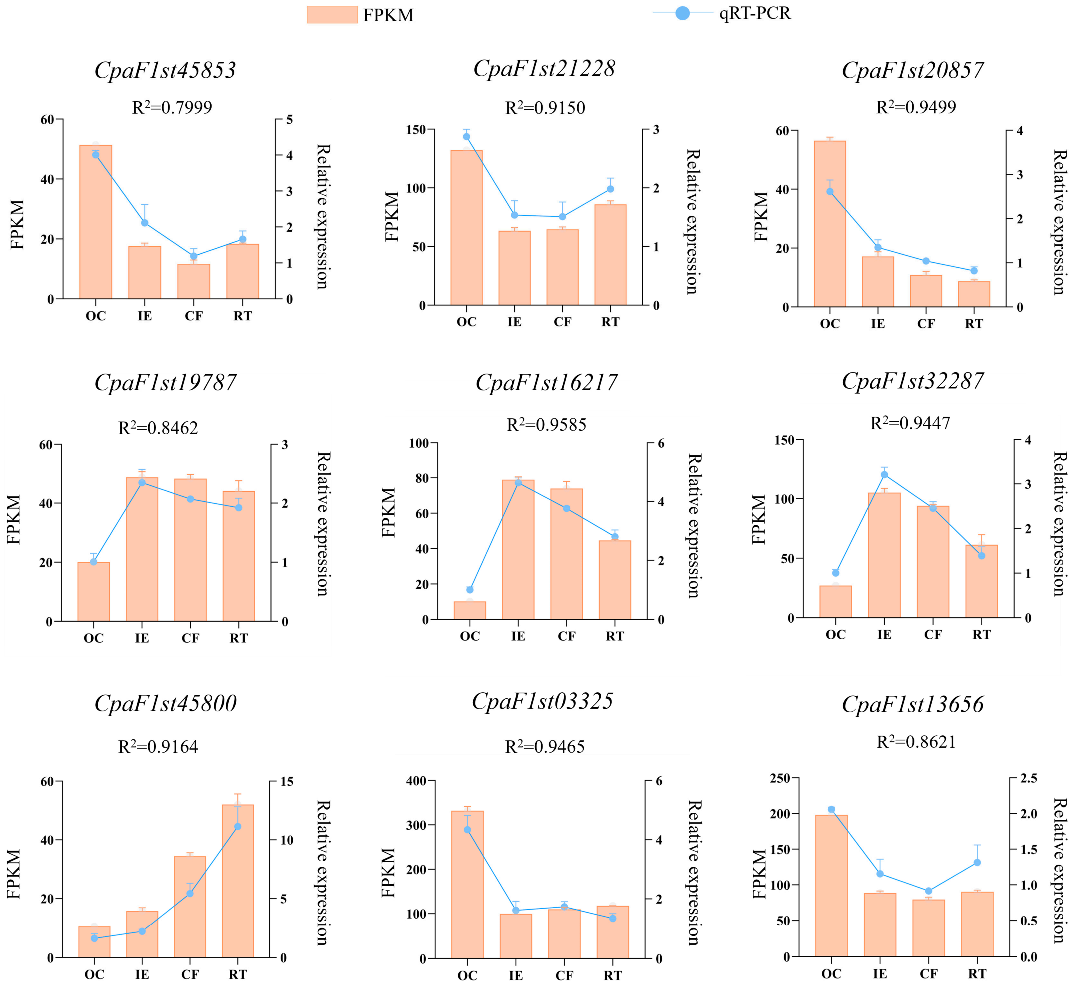

Figure S2. qRT-PCR validation of the transcriptome data results for 9 selected genes

Relative expression levels of qRT-PCR were calculated using 18sRNA as a standard. The samples from the base of *C. paliurus* soft cuttings were collected at OC, IE, CF, and RT status and were used for qRT-PCR analysis, while normalized gene levels in original cuttings were arbitrarily set to 1. OC: original cuttings; IE: cuttings in Initial expansion stage; CF: cuttings in callus formation stage; RT: cuttings in rooting stage.
